# Hospitals and the Approved Societies' Surplus

**Published:** 1921-02-19

**Authors:** S. R. Lamb

**Affiliations:** Secretary. The Sheffield Joint Hospitals' Council, 15 North Church Street, Sheffield


*To the Editor of* THE HOSPITAL.

February 14, 1921.

### Hospitals and the Approved Societies' Surplus.

*To the Editor of* THE HOSPITAL.

SIR,—Saturday's issue (February 12) of the *Manchester Guardian* intimates that a surplus of £8,000,000 is expected as the result of the Approved Societies' quinquennial valuation under the National Health Insurance Act. This handsome surplus is to be utilised partly in the form of increased sick benefit or in the maintenance of Approved Societies' members in hospitals, convalescent homes, and other essential medical services. No doubt Hospital Boards throughout the country will get into early touch with Friendly Societies' Councils to offer institutional treatment where possible.—I beg to be, Sir, your obedient servant,

S. R. LAMB, Secretary.

The Sheffield Joint Hospitals' Council,
15 North Church Street, Sheffield,
February 14, 1921.

